# Evaluating the Diagnostic and Prognostic Value of Interleukin-6 (IL-6) and Soluble Triggering Receptor Expressed on Myeloid Cells-1 (sTREM-1) in Systemic Inflammatory Response Syndrome (SIRS) and Sepsis in Adults

**DOI:** 10.7759/cureus.73310

**Published:** 2024-11-08

**Authors:** Eka Khuchua, Tamar Didbaridze, Giorgi Ormotsadze, Tamar Sanikidze, Elene Pachkoria, Levan Ratiani, Nino Gvajaia, Vasil Kupradze

**Affiliations:** 1 Department of Anesthesiology and Reanimatology, The First University Clinic of Tbilisi State Medical University, Tbilisi, GEO; 2 Department of Microbiology, The First University Clinic of Tbilisi State Medical University, Tbilisi, GEO; 3 Department of Problem of Radiation Safety, Ivane Beritashvili Center of Experimental Biomedicine, Tbilisi, GEO; 4 Department of Physics, Biophysics, Biomechanics and Informational Technologies, Tbilisi State Medical University, Tbilisi, GEO; 5 Department of Infectious Diseases, The First University Clinic of Tbilisi State Medical University, Tbilisi, GEO; 6 Department of Critical Care Medicine, Tbilisi State Medical University, Tbilisi, GEO; 7 Department of Medicine, Tbilisi State Medical University, Tbilisi, GEO

**Keywords:** apache-ii score, sepsis markers, sepsis prognosis, sepsis progression, systemic inflammatory response syndrome (sirs)

## Abstract

Introduction: Sepsis and systemic inflammatory response syndrome (SIRS) are significant concerns in intensive care units and contribute significantly to patient mortality. Traditional diagnostic markers such as C-reactive protein (CRP) and procalcitonin (PCT) often lack the sensitivity and specificity needed for early diagnosis and prognosis. Consequently, more reliable biomarkers are needed. This study aimed to evaluate interleukin-6 (IL-6) and soluble triggering receptor expressed on myeloid cells-1 (sTREM-1) as potential biomarkers to improve diagnostic and prognostic capabilities in sepsis and SIRS.

Methods: The study comprised 64 patients diagnosed with sepsis and SIRS, admitted to the Critical Care Department of the First University Clinic (Tbilisi, Georgia). Changes in the levels of CRP, PCT, IL-6, sTREM-1, and lactate were monitored over a five-day observation period, with measurements taken on days 0, 1, 2, 3, and 5.

Results: We found a significant logarithmic relationship between sTREM-1 levels and Acute Physiology and Chronic Health Evaluation II (APACHE II) scores (r = 0.922, p < 0.001), suggesting that sTREM-1 could serve as a valuable biomarker for early risk stratification in sepsis. Furthermore, sTREM-1 exhibited strong correlations with IL-6 and lactate levels, underscoring its potential as a predictor of disease severity. While CRP and PCT were more useful in tracking disease progression over time, their baseline levels were less predictive of early outcomes.

Conclusion: Our findings highlight the potential of sTREM-1, IL-6, and lactate as early diagnostic and prognostic markers in sepsis, where sTREM-1 is a critical biomarker for identifying high-risk patients. Further studies with larger cohorts are required to validate these results and explore the sTREM-1 clinical utility in guiding therapeutic interventions in sepsis management.

## Introduction

Sepsis and systemic inflammatory response syndrome (SIRS) are frequent contributors to mortality in intensive care units, with sepsis causing over 11 million deaths annually worldwide, accounting for approximately 20% of global deaths [1]. This emphasizes the critical need for early, accurate diagnosis and timely initiation of appropriate treatment, which is crucial for improving patient outcomes. In 1992, the Society for Critical Care Medicine (SCCM) introduced criteria for diagnosing SIRS and grading sepsis severity, including severe sepsis and septic shock [2]. However, these criteria had several fundamental limitations, such as an over-reliance on non-specific inflammatory responses, failure to consider organ dysfunction as a critical marker of sepsis progression, and poor differentiation between sepsis and other inflammatory conditions [3].

In 2016, the Sepsis-3 definitions addressed these limitations by incorporating the Sequential Organ Failure Assessment (SOFA) score and eliminating the concept of severe sepsis, providing clinicians with more precise diagnostic tools [4]. However, diagnosing early sepsis remains challenging due to the non-specific nature of traditional biomarkers like procalcitonin (PCT), C-reactive protein (CRP), and lactate. These biomarkers lack the sensitivity and specificity required for accurate and timely diagnosis, especially in adult populations. This underscores the urgent need for improved diagnostic tools. Researchers are investigating new biomarkers such as interleukin-6 (IL-6) and soluble triggering receptor expressed on myeloid cells-1 (sTREM-1) for their potential as diagnostic and prognostic indicators in sepsis, offering hope for improved early detection and outcomes in adult populations.

IL-6 is a pro-inflammatory cytokine involved in the early stages of the immune response [5]. It is synthesized in response to bacterial and viral infections, setting it apart from other interleukins [5]. IL-6 has diverse functions, including its effects on both humoral and cellular adaptive immunity, enhancing the sensitivity of monocytes and neutrophils and boosting the cytotoxic activity of natural killer (NK) cells [6]. sTREM-1 is a receptor found on the surface of neutrophils and monocytes, playing a significant role in the body's immune response to infection [6]. When microbial components activate sTREM-1, pro-inflammatory cytokines - including interleukin-1 beta (IL-1β) and tumor necrosis factor-alpha (TNF-α) - are released, which exacerbates the inflammatory response [7]. Assessing sTREM-1 levels may aid in distinguishing between infectious and non-infectious causes of systemic inflammation, thereby assisting clinicians in making timely and appropriate therapeutic decisions [7].

A previous study on pediatric patients assessed the diagnostic value of IL-6 and sTREM-1 in sepsis. Elevated IL-6 levels were associated with a more severe progression of febrile illness in children. However, further investigation is required to ascertain whether sTREM-1 helps identify sepsis and SIRS in febrile children [8]. Limited data exist on their application in adult sepsis, particularly regarding their prognostic value.

This study aimed to determine the predictive value of sTREM-1 in sepsis progression among adult patients by analyzing its correlation with Acute Physiology and Chronic Health Evaluation II (APACHE II) scores. Additionally, we investigated the relationship between sTREM-1 and other essential biomarkers in sepsis, including IL-6, lactate, PCT, and CRP. By assessing the roles of sTREM-1 and these biomarkers in early risk stratification and monitoring their changes over time, we aimed to enhance our understanding of their utility in predicting sepsis outcomes.

## Materials and methods

This prospective observational study included 64 patients with fever and clinical suspicion of sepsis, aged 18 to 84, admitted to the Critical Care Department of the First University Clinic (Tbilisi, Georgia) between October 2022 and August 2023. The study focused on patients meeting the criteria for SIRS or sepsis, as defined by the American College of Chest Physicians/Society of Critical Care Medicine (ACCP/SCCM).

Patients were diagnosed with SIRS if they presented with a body temperature over 38.5°C and at least one additional abnormal clinical sign from the following: heart rate greater than 90 beats per minute; respiratory rate greater than 20 breaths per minute or partial pressure of carbon dioxide (PaCO₂) less than 32 mmHg; leukocyte count greater than 12,000 cells/μL or less than 4,000 cell/μL; or the presence of more than 10% immature white blood cells (bands).

Sepsis was identified when SIRS occurred in the presence of a suspected infection, along with an acute increase of 2 or more points on the SOFA score. The study further classified severe sepsis as sepsis complicated by organ dysfunction. The quick Sequential Organ Failure Assessment (qSOFA) score, which includes a respiratory rate of 22 or more breaths per minute, systolic blood pressure of 100 mmHg or less, and altered mental status, was also considered in evaluating the severity of sepsis.

Exclusion criteria for the study included patients under the age of 18, those undergoing chemotherapy or corticosteroid therapy, and those diagnosed with HIV, hepatitis B virus (HBV), or hepatitis C virus (HCV). The APACHE II score, which assesses disease severity, was calculated on the day of admission and monitored daily to track patient progression. Based on their APACHE II scores, patients were divided into two groups: those with scores indicating a mortality risk of less than 50% (APACHE II scores below 20) and those with scores indicating a mortality risk of 50% or more (APACHE II scores of 20 or higher). Routine medical examinations, including blood sampling, vital sign monitoring (heart rate, respiratory rate, and blood pressure), and clinical assessments of organ function, were performed by trained technicians under the supervision of attending physicians. The clinical laboratory team conducted all blood draws and sample preparations to ensure sample handling and analysis consistency.

Blood samples for biomarker analysis were collected at specific intervals: day 0 (upon admission) and days 1, 2, 3, and 5. These days were chosen to monitor the progression of sepsis and the patient's response to treatment over the first critical days of their ICU stay. Biomarker analyses included CRP, PCT, IL-6, sTREM-1, and lactate. Lactate was measured to assess the degree of metabolic stress and tissue hypoxia, critical in monitoring sepsis severity. IL-6 concentrations were measured using an electrochemiluminescence method on the Cobas e411 analyzer (Elecsys IL; Roche Diagnostics GmbH, Mannheim, Germany). The lower detection limit was 1.5 pg/mL. sTREM-1 levels were quantified using an enzyme-linked immunosorbent assay (ELISA) kit (Quantikine ELISA Test, Wuhan Fine Biotech, Wuhan, China), with a detection limit of 18.75 pg/mL. PCT levels were analyzed via an enzyme-linked fluorescent immunoassay, with a lower detection threshold of 3.88 pg/mL. The upper reference range for healthy subjects for PCT was 0.05 ng/mL. CRP and lactate were measured using standard biochemical assays.

To identify potential causal relationships between studied characteristics, Pearson's correlation coefficient (Pearson r) and linear regression methods were employed; the statistical significance was assessed using a p-value threshold. Factorial analysis of variance (ANOVA) and repeated measures ANOVA were used for the evaluation of the statistical significance between study groups. Statistical Package for the Social Sciences (IBM SPSS Statistics for Windows, IBM Corp., Version 19.0, Armonk, NY) was used to perform calculations and visualize results.

The study was conducted in accordance with the ethical principles outlined in the Declaration of Helsinki and received approval from the Institutional Review Board (IRB) of the Ethics Committee at Tbilisi State Medical University (IRB number N8-2021/92). Patient confidentiality was upheld, and data anonymity was ensured throughout the course of the study.

## Results

Initially, 236 patients were admitted to the ICU with fever and suspected sepsis. After applying the inclusion and exclusion criteria, 64 patients met the final eligibility requirements and were selected to participate in the study.

Our study explored the relationship between APACHE II scores and sTREM-1 levels in the progression of sepsis. To facilitate this, we categorized the patients into two groups based on their APACHE II scores. The first group, comprising 38 patients (59%), included those with APACHE II scores below 20, indicating a mortality risk of less than 50%. The second group consisted of 26 patients (41%) with APACHE II scores of 20 or higher, indicating a mortality risk more significant than 50%. The demographic characteristics of both groups are detailed in Table 1.

**Table 1:** Demographic data APACHE II: Acute Physiology and Chronic Health Evaluation II

	APACHE II < 50% (n=38)	APACHE II > 50% (n=26)	Overall (n=64)
Age (years)			
Median (Min, Max)	51 (25, 84)	45 (18, 76)	48 (18, 84)
Gender			
Male	11 (28.9%)	9 (34.6%)	20 (31.3%)
Female	27 (71.1%)	17 (65.4%)	44 (68.7%)
Hospitalization Days			
Mean ± SD	8.5 ± 3.2	12 ± 4.1	10 ± 3.8

**Figure 1:**
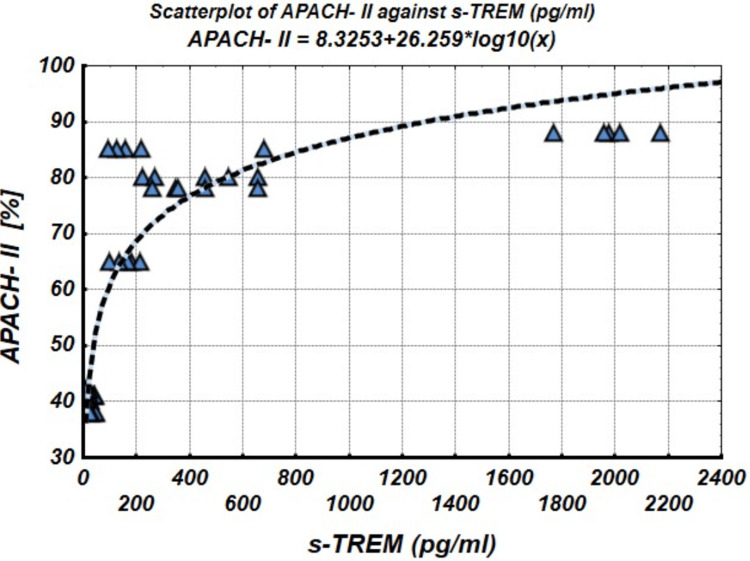
APACHE-II/sTREM-1 scattergram and logarithmic function interpolation curve APACHE-II: Acute Physiology and Chronic Health Evaluation-II; sTREM-1: soluble triggering receptor expressed on myeloid cells-1

To explore the relationship between APACHE II scores and sTREM-1 levels, we analyzed their scatter plot in Figure 1. The scatter plot shows a clear logarithmic relationship between the two variables, confirmed by linear regression analysis. The equation derived from the analysis is APACHE-II = a + b* Log_10_(sTREM-1).

**Figure 2:**
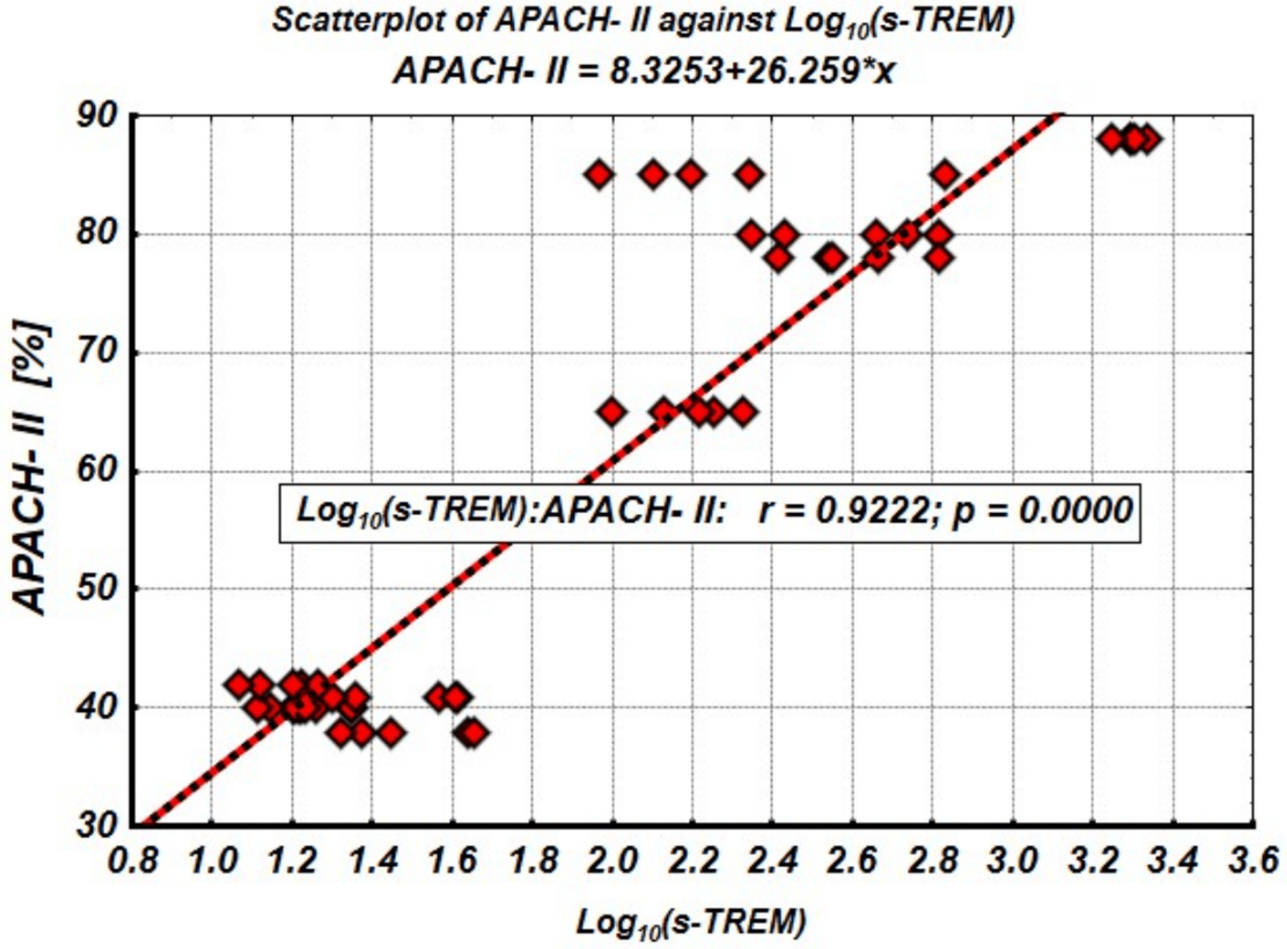
APACHE-II/Log10(sTREM-1) scattergram and linear regression APACHE-II: Acute Physiology and Chronic Health Evaluation-II; sTREM-1: soluble triggering receptor expressed on myeloid cells-1

The intercept coefficient was 8.325 (standard error = 3.310, p = 0.015), and the slope coefficient was 26.259 (standard error = 1.580, p < 0.001). The correlation coefficient between APACHE-II and Log₁₀(sTREM-1) was r = 0.922, with a significance level of p < 0.001, as shown in Figure 2. This strong correlation indicates that as sTREM-1 levels increase, APACHE-II scores increase, initially sharply and then plateauing due to a saturation effect. These findings suggest that sTREM-1 could serve as a valuable biomarker in predicting the progression of sepsis, with Log₁₀(sTREM-1) emerging as a highly informative predictor in sepsis progression.

**Figure 3:**
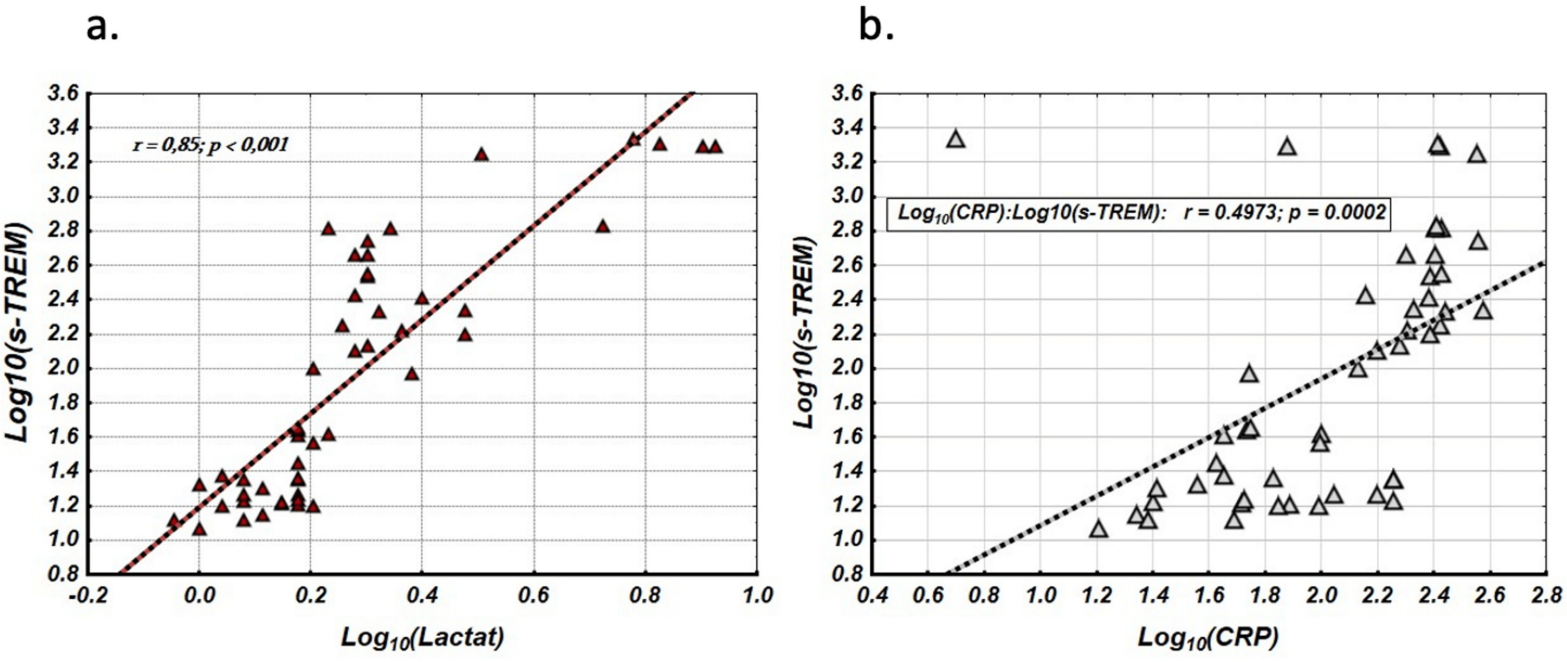
(a) Log10(sTREM-1)/Log10(Lactate) scatterplot and linear regression; (b) Log10(sTREM-1)/Log10(CRP) scatterplot and linear regression sTREM-1: soluble triggering receptor expressed on myeloid cells-1; CRP: C-reactive protein

We further analyzed the relationship between Log₁₀(sTREM-1) and other serum parameters, including IL-6, PCT, CRP, and lactate. Table 2 presents the correlation coefficients, along with their statistical significance, between Log₁₀(sTREM-1) and these biomarkers. All studied characteristics, except leukocytes (r = 0.152; p = 0.295), showed significant correlations with Log₁₀(sTREM-1). Two distinct patterns emerged from this analysis. The correlation reliability for IL-6 and lactate, with Log_10_(sTREM-1) strengthened after logarithmic transformation (increased from r = 0,630, p < 0,001 to r = 0,857, p < 0,001). Similarly, the correlation for lactate increased from r = 0.750 (p < 0.001) to r = 0.851 (p < 0.001) as illustrated in Figure 3a. In contrast, the correlation with Log₁₀(sTREM-1) weakened after transformation for PCT and CRP. The correlation for PCT decreased from r = 0.722 (p < 0.001) to r = 0.680 (p < 0.001), while the correlation for CRP decreased from r = 0.661 (p < 0.001) to r = 0.493 (p < 0.001), as shown in Figure 3b. These findings suggest that the regulatory mechanisms involving sTREM-1, IL-6, and lactate are more closely associated with the early risk of developing sepsis than those involving PCT and CRP.

**Table 2:** Correlations variables/Log10(sTREM-1); marked correlations are significant at p < 0.050 sTREM-1: soluble triggering receptor expressed on myeloid cells-1; CRP: C-reactive protein

Variables	Log_10_(sTREM)	
	Correlation (r)	p-value
Log_10_(sTREM)	r=1.00	
Leucocyte’s	r=0.152	p=0.295
Procalcitonin	r=0.722	p<0.001
Log_10_(Procalcitonin)	r=0.680	p<0.001
Interleukin	r=0.630	p<0.001
Log_10_(Interleukin)	r=0.857	p<0.001
CRP (mg/L)	r=0.661	p<0.001
Log_10_(CRP)	r=0.493	p<0.001
Lactate (mmol/L)	r=0.750	p<0.001
Log_10_(Lactate)	r=0.851	p<0.001
Lactate (mmol/L)	r=0.750	p<0.001
Log_10_(Lactate)	r=0.851	p<0.001

To further elucidate these relationships, we analyzed the dynamics of the logarithmic values of the biomarkers on days 0, 1, 3, and 5 of observation. A Repeated Measures Analysis of Variance (RM-ANOVA) was applied. The APACHE-II scores were considered as a between-group factor, categorized into two levels: high risk (APACHE-II ≥ 50%) and low risk (APACHE-II < 50%) of lethality. The logarithmic values of the biomarkers - Log₁₀(sTREM-1), Log₁₀(Lactate), and Log₁₀(Interleukin) - measured on days 0, 1, 3, and 5 were treated as repeated measures factors with five levels. Clinically, higher log-transformed values represent substantial increases in actual biomarker levels, indicating greater disease severity.

**Figure 4:**
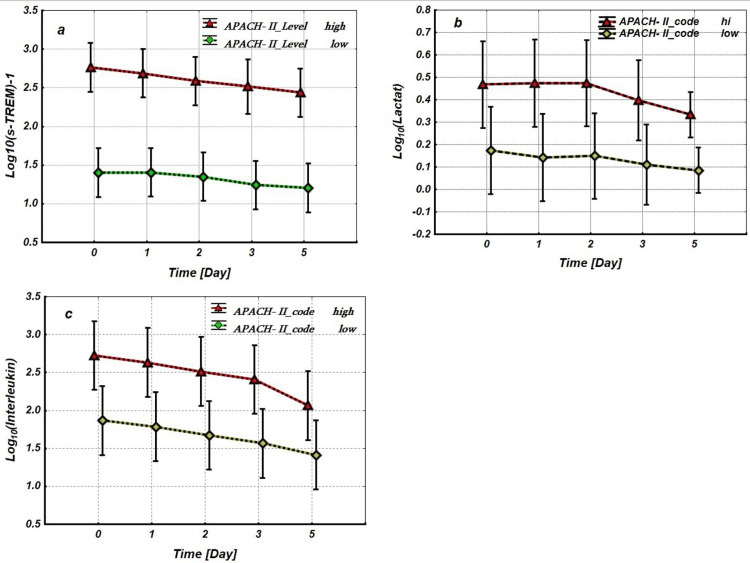
Repeated measures analysis of variance. Between-group factor - APACHE-II scores (with 2 levels: high (APACHE-II ≥ 50%) and low (APACHE-II < 50%) risk of lethality), repeated measure factors (within effects). (a) Log10(sTREM-1), (b) Log10(Lactate) and (c) Log10(Interleukin) (with 5 levels: 0, 1, 2, 3 and 5 days) - Log_10_(sTREM-1) vs. APACHE-II scores, factors interaction effect: F=0.26; p=0.89. - Log_10_(Lactate) vs. APACHE-I scores, factors interaction effect: F=0.403; p=0.80. - Log_10_(Interleukin) vs. APACHE-II scores, factors interaction effect: F=0.41; p=0.82. - Between-group factors (Log_10_(sTREM-1) vs. APACHE-II scores) effects (Time - 0 Day): F=47.2 p<0.001 - Between-group factors (Log_10_(Lactate) vs. APACHE-II scores) effects (Time - 0 Day): F=6.10, p=0.034. - Between-group factors (Log_10_(Interleukin) vs. APACHE-II scores) effects (Time - 0 Day): F=11.35, p=0.01 APACHE-II: Acute Physiology and Chronic Health Evaluation-II; sTREM-1: soluble triggering receptor expressed on myeloid cells-1

As shown in Figure 4, the levels of Log₁₀(sTREM-1), Log₁₀(lactate), and Log₁₀(IL-6) on day 0 were significantly higher in the unfavorable prognosis group compared to the favorable prognosis group. This pattern does not change over the observation period (0-5 days), as indicated by the statistically unreliable interaction effect between the between-group factor (APACHE-II scores) and the Repeated measure factors (Log₁₀(sTREM-1), Log₁₀(lactate), and Log₁₀(IL-6)) (Figure 4).

**Figure 5:**
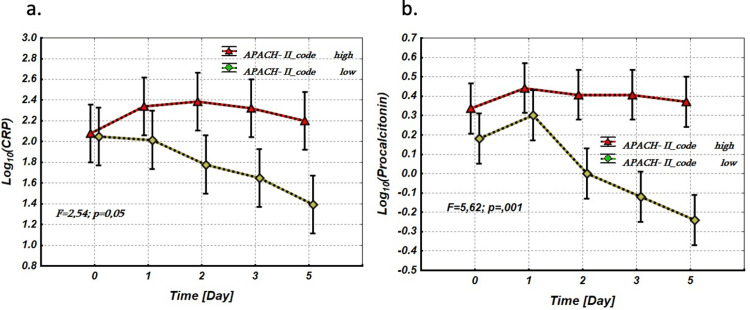
Repeated measures analysis of variance. Between-group factor - APACHE-II scores (with 2 levels: high (APACHE-II ≥ 50%) and low (APACHE-II < 50%) risk of lethality), repeated measure factors (within effects). (a) Log10(CRP) and (b) Log10(Procalcitonin) (with 5 levels: 0, 1, 2, 3 and 5 days) - Log_10_(CRP) vs. APACHE-II scores factors interaction effect: F=2.58; p=0.038. - Log_10_(Procalcitonin) vs. APACHE-II factors interaction effect: F=5.19; p=0.002. - Between-group factors (Log_10_(CRP) vs. APACHE-II scores) effects (Time - 0 Day): F=0.007, p=0.93 - Between-group factors (Log_10_(Procalcitonin) vs. APACHE-II scores) effects (Time - 0 Day): F=2.07, p=0.053 APACHE-II: Acute Physiology and Chronic Health Evaluation-II; CRP: C-reactive protein

In contrast, the initial levels of CRP and PCT on day 0 did not show significant differences between the between-group factors (Figure 5). However, their dynamics over time differed significantly between patients with favorable and unfavorable prognoses, as depicted in Figure 5. This indicates that CRP and PCT levels are more reflective of disease severity and progression rather than initial prognosis.

Our study indicates that sTREM-1, lactate, and IL-6 are closely associated with sepsis severity and may serve as early predictive biomarkers for unfavorable prognosis. In contrast, CRP and PCT levels provide valuable information for assessing disease severity and monitoring progression over time. This distinction is crucial for tailoring patient management strategies and improving clinical outcomes in sepsis care.

## Discussion

In our study, we confirmed that sTREM-1 could serve as a reliable predictor of sepsis progression, as indicated by the clear logarithmic relationship between APACHE II scores and Log₁₀(sTREM-1) levels. Higher sTREM-1 levels are strongly associated with higher APACHE II scores, reflecting the increasing severity of illness and higher risk of mortality in sepsis patients. This finding is consistent with emerging evidence on the diagnostic value of sTREM-1 in sepsis. For instance, a study demonstrated that sTREM-1 had a high sensitivity of 0.80 in predicting 28-day mortality in sepsis [9]. Our study supports these findings, as we observed a strong positive correlation between sTREM-1 and APACHE II scores (r = 0.922, p < 0.001). Additionally, the previous study showed that sTREM-1 and APACHE II scores were higher in sepsis patients than those with SIRS [10], aligning with our conclusion that sTREM-1 is closely linked to the progression of sepsis.

Our study demonstrated a significant correlation between sTREM-1 levels and other commonly used sepsis biomarkers, including IL-6, lactate, PCT, and CRP. IL-6 and lactate, in particular, exhibited the strongest correlations with sTREM-1, especially after applying a logarithmic transformation, indicating a more pronounced relationship with sepsis risk. These findings are consistent with existing literature emphasizing the roles of IL-6 and lactate in sepsis pathophysiology and prognosis. Research has shown that IL-6 is closely linked to severity scores such as APACHE II and SOFA, serving as a critical indicator of poor outcomes in sepsis. Patients with IL-6 levels of 100 pg/mL or higher had nearly three times the odds of 28-day mortality compared to those with lower levels, highlighting IL-6's critical prognostic value [11]. Similarly, elevated lactate levels, particularly above four mmol/L, have long been associated with higher mortality in sepsis patients, further reinforcing its importance as a prognostic marker [12].

In contrast, the correlations between sTREM-1 and PCT and CRP weakened after logarithmic transformation. This indicates that PCT and CRP may offer more limited predictive value for the early identification of sepsis risk. These findings align with prior studies demonstrating that CRP and PCT are more indicative of disease severity than early indicators of poor outcomes [13]. One study found that both CRP and PCT had limited utility in predicting 30-day all-cause mortality, with no significant association between these biomarkers and mortality risk in patients with sepsis or septic shock [5]. PCT is mainly recognized as a marker of bacterial infection and is frequently used to guide antibiotic therapy. However, its ability to predict mortality or sepsis progression is less robust than other markers, such as sTREM-1 or IL-6 [14].

Our findings also align with studies investigating the dynamics of sTREM-1 and other biomarkers over time [15-17]. We found that initial levels of sTREM-1, IL-6, and lactate on day 0 were significantly higher in patients with an unfavorable prognosis than those with a favorable prognosis. These biomarkers can be valuable for early risk stratification in sepsis. However, their levels throughout the observation period did not differ significantly between the prognosis groups, implying that initial measurements are more indicative of prognosis than subsequent changes. This aligns with previous research showing that early sTREM-1 levels, measured within 24 hours of sepsis onset, effectively predict sepsis severity and 28-day mortality, while changes in sTREM-1 levels during follow-up provide limited additional prognostic value [18].

In contrast, while CRP and PCT did not show significant differences at baseline, their dynamics over time were markedly different between patients with favorable and unfavorable prognoses. This supports previous findings that CRP and PCT are better suited for monitoring disease severity and response to treatment rather than for early prognostication [15]. Therefore, sTREM-1, IL-6, and lactate should be considered in early sepsis risk assessment, while CRP and PCT may be more beneficial for tracking clinical progression.

Our use of log-transformed values for sTREM-1, IL-6, and other biomarkers allowed for a clearer statistical analysis and interpretation. For clinical interpretation, an increase in the log value reflects a marked rise in the biomarker's actual concentration, underscoring the severity of the condition.

It is important to note that our study was conducted on a relatively small cohort of 64 patients. Therefore, while the clinical efficacy of the biomarkers discussed in this article is evident, the outcomes of our study may not be fully representative of the general population. Generalizing the complex parameters determined from this cohort may be less appropriate due to its probable low representativeness. However, our findings provide a solid foundation for future investigations. In particular, increasing the size of the studied cohort in future studies will allow us to assess the representativeness of our results and their generalization to other populations. Further studies with larger cohorts are needed to validate these findings and explore sTREM-1 as a therapeutic target.

In summary, our study emphasizes the significance of sTREM-1 as a critical biomarker for early risk stratification in sepsis, demonstrating strong correlations with APACHE-II scores and key biomarkers such as IL-6 and lactate. These findings support its potential for early identification of high-risk patients. In contrast, CRP and PCT are more suited for monitoring disease progression, underscoring the importance of a multifaceted approach to sepsis management that incorporates both early prognostic markers and ongoing monitoring of clinical dynamics.

## Conclusions

Our study highlights sTREM-1 as a valuable biomarker for predicting sepsis severity and progression, as evidenced by its strong correlation with APACHE II scores and its association with other key markers, such as IL-6 and lactate. These findings suggest that sTREM-1 could play a critical role in sepsis's early diagnosis and risk stratification. In contrast, while CRP and PCT did not show significant predictive value at baseline, their dynamic changes over time indicated disease severity and response to treatment, underscoring their utility in monitoring sepsis progression. Further research is necessary to validate the clinical application of sTREM-1 and to explore its potential in guiding therapeutic interventions in sepsis management.
